# Trends in Dementia Risk vs Cognitive Function: Rural, Micropolitan & Urban Areas

**DOI:** 10.1093/geroni/igaf122.1599

**Published:** 2025-12-31

**Authors:** Ladan Ghazi Saidi

**Affiliations:** University of Nebraska Kearney, Kearney, Nebraska, United States

## Abstract

Cognitive aging is influenced by environmental, demographic, and healthcare factors. Rural populations may face a higher risk of cognitive decline due to healthcare access and socioeconomic conditions. We assessed cognitive function across rural, micropolitan, and urban populations in the United States using the Montreal Cognitive Assessment (MoCA) in a sample of 150 adults aged 55 and older. Participants were clustered into rural (<5,000 residents, n = 50), micropolitan (5,000–50,000 residents, n = 50), and urban (>50,000 residents, n = 50). Statistical analyses included descriptive statistics and independent samples t-tests, with significance set at p < 0.05. The overall MoCA score averaged 25.46 (SD = 2.88, range = 14–30), aligning with prior research on aging populations and mild cognitive impairment thresholds. While no significant differences were observed in MoCA scores across rural (M = 25.52, SD = 3.124), micropolitan (M = 25.33, SD = 2.987), and urban (M = 25.58, SD = 2.663) groups, the proportion of participants scoring below the MoCA cutoff (<26) followed a trend of rural (43.3%) > micropolitan (38.5%) > urban (33.3%). Age distributions varied, with micropolitan participants being the oldest (M = 69.82 years) compared to rural (M = 67.2 years) and urban (M = 66.41 years) participants. Although MoCA scores did not significantly differ by residential classification, the higher proportion of rural participants scoring below the cutoff suggests an increased risk for cognitive impairment. Findings highlight the need for further research into modifiable dementia risk factors to inform policy development regarding healthcare access, education, and lifestyle differences.

